# The cardiac atlas project: rationale, design and preliminary results

**DOI:** 10.1186/1532-429X-13-S1-O72

**Published:** 2011-02-02

**Authors:** Pau Medrano-Gracia, Michael Backhaus, David A Bluemke, Jae Do Chung, Brett R Cowan, Paul J Finn, Carissa G Fonseca, Peter J Hunter, Alan H Kadish, Daniel C Lee, Joao AC Lima, Kalyanam Shivkumar, Wenchao Tao, Alistair A Young

**Affiliations:** 1 The University of Auckland, Auckland, New Zealand; 2 Department of Radiology, Johns Hopkins Hospital, Baltimore, MD, USA; 3 Diagnostic CardioVascular Imaging, UCLA, Los Angeles, CA, USA; 4 Bluhm Cardiovascular Institute, Northwestern Memorial Institute, Chicago, IL, USA; 5 UCLA Cardiac Arrhythmia Center, Los Angeles, CA, USA

## Objective

To develop a statistical map of regional wall motion in healthy and diseased populations using a standardized database of cardiovascular magnetic resonance studies.

## Background

The Cardiac Atlas Project (CAP) is a NIH sponsored international collaboration to establish a web-accessible structural and functional atlas of the normal and pathological heart as a shared resource for the clinical, research and educational communities.

## Methods

Images, derived ventricular contours, and clinical text data have been contributed from several studies. To date, 2864 cases have been contributed from the MESA study [1] comprising asymptomatic volunteers, and 470 cases have been contributed from the DETERMINE study [2] comprising patients with myocardial infarction. DICOM images were de-identified using HIPAA compliant software [3]. Only those cases with informed consent and IRB approval compatible with data sharing were included. To illustrate the potential of this resource, a preliminary statistical analysis was performed on a subset of 300 cases from DETERMINE and 200 cases from MESA. A finite element model of the left ventricle was customized to each case using a standardized mapping which registered each anatomical location within a standard coordinate system. Shape and motion distributions were quantified across cohorts using principal component analysis and multidimensional statistical tests.

## Results

**Figure 1:**
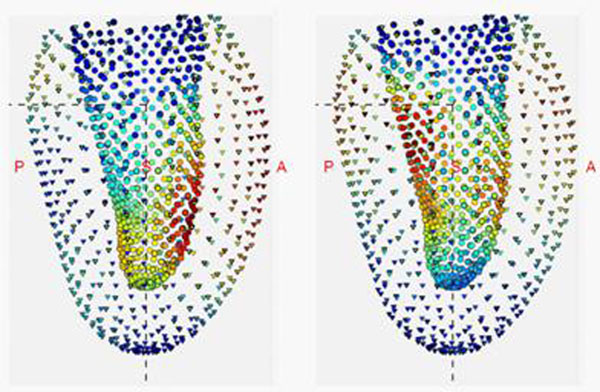
Regions of statistical difference between MESA (asymptomatic) and DETERMINE (myocardial infarction) subgroups. Left: antero-septal infarct DETERMINE subgroup. Right: infero-posterior infarct DETERMINE subgroup. Colours indicate Hotelling T^2^ p-values, blue least significant, red most significant. Viewpoint is from the septum with the posterior wall to the left. Triangles show epicardial points, and circles show endocardial points, sampled from the average MESA geometry at end systole.

The analysis automatically determined the major characteristics and statistical distribution of shape and motion in the MESA and DETERMINE groups. The main modes were associated with well-known clinical indices of cardiac remodelling including size, sphericity and mitral valve geometry. The Hotelling T^2^ test showed significant differences between the MESA cohort and subgroups of the DETERMINE cohort, stratified according to infarct location (Figure 1).

## Conclusion

Standardized mapping of shape and motion facilitates statistical characterization of cardiac performance, providing a powerful resource for the scientific community. Applications for use of the resource can be made from the website (http://www.cardiacatlas.org).
